# Non-traditional lipid biomarkers in atherosclerotic cardiovascular disease: pathophysiological mechanisms and strategies to address residual risk

**DOI:** 10.3389/fendo.2025.1576602

**Published:** 2025-07-10

**Authors:** Cong Wang, Haifeng Fu, Hao Xu, Handong Yang, Xinwen Min, Wenwen Wu, Zhixin Liu, Dongfeng Li, Yun Dong, Jun Chen

**Affiliations:** ^1^ Sinopharm Dongfeng General Hospital (Hubei Clinical Research Center of Hypertension), Hubei Key Laboratory of Wudang Local Chinese Medicine Research, Hubei University of Medicine, Shiyan, Hubei, China; ^2^ Shiyan Key Laboratory of Virology, Hubei University of Medicine, Shiyan, China; ^3^ School of Public Health, Hubei University of Medicine, Shiyan, Hubei, China

**Keywords:** atherosclerosis, cardiovascular disease, residual risk, blood lipids, biomarkers

## Abstract

Atherosclerotic cardiovascular disease (ASCVD) pathogenesis is fundamentally driven by dyslipidemia, characterized by lipid metabolism disorders that facilitate cholesterol deposition within damaged vascular endothelia. This process culminates in atherosclerotic plaque formation and coronary stenosis, ultimately inducing myocardial ischemia. While low-density lipoprotein cholesterol (LDL-C) remains the principal lipid determinant of ASCVD progression, emerging evidence indicates persistent residual cardiovascular risk despite optimal statin-mediated LDL-C control. This review aims to systematically evaluate the contributory role of non-traditional lipid biomarkers in ASCVD pathophysiology and clinical outcomes. Through comprehensive analysis of current research, we examine the biological properties and atherogenic mechanisms of non-conventional lipid particles, epidemiological evidence linking these biomarkers with residual cardiovascular risk, and therapeutic implications of targeting alternative lipid pathways. Particular emphasis is placed on elucidating the pathophysiological interplay between triglyceride-rich lipoproteins, lipoprotein(a), and oxidized phospholipids with vascular inflammation and plaque instability. Furthermore, we critically appraise recent clinical trial data regarding novel lipid-modifying agents and propose future research directions to address current knowledge gaps in residual risk management. This synthesis underscores the necessity of expanding therapeutic strategies beyond LDL-C reduction to achieve comprehensive cardiovascular risk mitigation.

## 1 Introduction

Cardiovascular diseases (CVD) are among the leading chronic illnesses that threaten human life and health on a global scale, attracting significant attention from the medical community. CVD encompasses a wide range of heart and vascular disorders, including coronary artery disease, heart failure, arrhythmia, valvular heart disease, and peripheral artery disease (1–4). Among these various types of CVD, atherosclerotic cardiovascular disease (ASCVD) has garnered particular interest due to its high morbidity and mortality rates. According to relevant statistics in China, ASCVD has emerged as the primary cause of death among both urban and rural populations, with ischemic heart disease and ischemic stroke being the predominant types (5). From a pathological perspective, ASCVD primarily manifests as coronary artery disease, cerebrovascular disease, and peripheral artery disease, with its core pathogenesis closely related to the formation and progression of atherosclerotic plaques and the subsequent pathophysiological changes they induce. These pathological alterations have a direct impact on patient health. For instance, coronary artery obstruction can lead to angina or myocardial infarction, carotid artery disease may result in ischemic stroke, and peripheral artery disease can cause insufficient perfusion of the (6, 7). Among the various biomarkers associated with cardiovascular disease, low-density lipoprotein cholesterol (LDL-C) is widely recognized as a key predictor and a primary target of lipid-lowering therapy, as it is considered a major trigger of atherogenesis (8–10). However, clinical studies have demonstrated that, even with pharmacological interventions that effectively lower LDL-C levels, a significant residual cardiovascular risk remains (11–13). In recent years, increasing evidence has highlighted the potential role of several nontraditional lipid components-such as lipoprotein(a) [Lp(a)], apolipoprotein B (ApoB), non-high-density lipoprotein cholesterol (Non-HDL-C), and remnant cholesterol (RC)-in contributing to this residual risk (14, 15). These lipid components, independent of LDL-C, may play a crucial role in the development and progression of atherosclerosis, offering new research directions and potential therapeutic targets for the prevention and management of cardiovascular diseases.

## 2 The known relationship between conventional lipids and ASCVD

The prevention and management of hypercholesterolemia have emerged as major public health concerns, given its strong association with an elevated risk of atherosclerotic cardiovascular disease (ASCVD). Substantial evidence underscores the critical role of maintaining normal cholesterol levels in both primary and secondary prevention of ASCVD (16, 17). From a pathophysiological perspective, elevated cholesterol levels alter the permeability of the arterial endothelium, facilitating the infiltration of lipid components-particularly low-density lipoprotein cholesterol (LDL-C)—into the arterial wall (18). Upon exposure to oxidative stress, LDL undergoes structural modifications, primarily through oxidative alterations of its apolipoprotein B (ApoB) component. These oxidized LDL particles are subsequently recognized and internalized via scavenger receptors on macrophages, leading to their transformation into foam cells (19–21). This process is a fundamental driver of atherosclerosis, wherein elevated plasma LDL-C levels play a pivotal role in the initiation, progression, and destabilization of atherosclerotic plaques (19, 22–25). Notably, epidemiological and clinical studies have consistently identified elevated LDL-C as one of the most significant modifiable risk factors for ASCVD.

Conversely, high-density lipoprotein (HDL) exerts atheroprotective effects through multiple mechanisms (26). Firstly, HDL enhances endothelial function and preserves vascular homeostasis by stimulating nitric oxide (NO) production and activating endothelial nitric oxide synthase (eNOS). Secondly, apolipoprotein A-I (ApoA-I), the principal protein constituent of HDL, plays a key role in reverse cholesterol transport (RCT), facilitating the efflux of cholesterol from peripheral tissues to the liver for metabolism and excretion. This process effectively reduces lipid accumulation within the vascular wall. Furthermore, HDL mitigates inflammation and oxidative stress, thereby attenuating the onset and progression of atherosclerosis. Specifically, HDL promotes cholesterol efflux from macrophages and foam cells by regulating the expression of ATP-binding cassette transporters A1 (ABCA1) and G1 (ABCG1), further reinforcing its protective role (27–29).

In summary, total cholesterol (TC) and LDL-C contribute substantially to the development and progression of atherosclerosis through mechanisms such as arterial lipid deposition, oxidative modification, and subsequent inflammatory responses leading to plaque formation. In contrast, HDL-C confers protective effects via RCT, anti-inflammatory and antioxidant properties, and endothelial function enhancement. The dynamic interplay between these pro- and anti-atherogenic processes serves as a fundamental regulatory mechanism in atherosclerosis and provides a critical theoretical foundation for targeted interventions in ASCVD prevention and treatment.

## 3 The concept of non-traditional lipids and their potential role in ASCVD

In the risk assessment of atherosclerotic cardiovascular disease (ASCVD), non-traditional lipid markers serve as a valuable complement to conventional lipid testing, offering a more comprehensive perspective. These markers primarily include apolipoprotein B (ApoB), lipoprotein(a) [Lp(a)], non-high-density lipoprotein cholesterol (Non-HDL-C), remnant cholesterol (RC), and non-fasting lipids. In addition, key lipid markers such as oxidized low-density lipoprotein (oxLDL), small dense low-density lipoprotein (sdLDL), and apolipoprotein C-III (ApoC-III) have also garnered significant attention (30, 31).

Although clinical studies have demonstrated that therapeutic strategies targeting low-density lipoprotein cholesterol (LDL-C) can effectively reduce ASCVD risk, many individuals with normal or even low LDL-C concentrations still experience ASCVD events or progression of atherosclerosis (11, 32, 33). This residual risk suggests that relying solely on LDL-C levels may not provide a comprehensive assessment of a patient’s cardiovascular risk. Non-traditional lipid markers offer critical insights beyond conventional lipid testing, enabling more precise identification of high-risk populations and providing a theoretical foundation for personalized treatment. This review systematically discusses the major types of these non-traditional lipid markers, including Non-HDL-C, ApoB, Lp(a), and RC, with the aim of introducing new perspectives for ASCVD risk assessment and prevention.

## 4 Biological mechanisms of non-traditional blood lipids and ASCVD

### 4.1 Non-HDL-C

Non-HDL-C represents the total cholesterol content in all lipoprotein particles except HDL-C, including LDL, VLDL, VLDL remnants, and lipoprotein(a) [Lp(a)]. These particles overlap functionally with traditional LDL-C pathways: LDL and VLDL remnants undergo the same ApoB-mediated receptor interactions and oxidative modifications that characterize LDL-C–driven atherogenesis (34, 35). Specifically, once Non-HDL-C particles penetrate the arterial intima—via retention by proteoglycans—they undergo oxidative modification analogous to LDL oxidation, producing ox-LDL (34). This shared pathway links Non-HDL-C to traditional LDL-C–mediated foam cell formation, macrophage activation, and NF-κB–dependent inflammatory cascades (19, 22). In addition, VLDL remnants contribute excess substrate for hepatic very-low-density lipoprotein secretion, thereby feeding back into LDL-C production and perpetuating the classical cholesterol-homeostasis feedback loop that modulates LDL receptor activity in the liver (18).

Once deposited beneath the endothelium, Non-HDL-C components [LDL, VLDL remnants, and Lp(a)] oxidize into ox-LDL and oxidized VLDL, both of which are recognized by macrophage scavenger receptors. The result is foam cell formation and secretion of proinflammatory cytokines (e.g., IL-6, TNF-α), reinforcing traditional inflammatory pathways that link oxidized LDL to NLRP3 inflammasome activation (19, 27). Elevated Non-HDL-C also exacerbates endothelial dysfunction by reducing endothelial nitric oxide synthase (eNOS) activity and stimulating expression of adhesion molecules (VCAM-1, ICAM-1), thereby reinforcing the classic LDL-C–endothelial interaction (26).

Moreover, Non-HDL-C promotes a prothrombotic milieu: Oxidized remnants and Lp(a) can bind to fibrinogen, inhibiting fibrinolysis—a mechanism also observed in LDL-C–mediated coagulation activation (36). Platelet activation by oxidized lipoproteins further augments thrombin generation, linking Non-HDL-C directly to the traditional LDL-C pathways of plaque destabilization and thrombosis (36).

Because cholesterol within LDL particles can be redistributed to VLDL remnants under high triglyceride conditions, Non-HDL-C serves as an indirect marker of total atherogenic particle burden—paralleling LDL particle number measurement (apolipoprotein B). Consequently, Non-HDL-C more accurately reflects the combined traditional LDL-C and VLDL‐derived cholesterol load, making it especially informative when classical LDL-C measurements underestimate residual risk in hypertriglyceridemic patients (37).

### 4.2 ApoB

Apolipoprotein B (ApoB) is a critical structural protein in atherogenic lipoproteins, with each atherogenic particle containing exactly one ApoB molecule. The two isoforms—ApoB48 and ApoB100—coexist, but ApoB100 [in VLDL, IDL, LDL, and Lp(a)] accounts for ~90% of circulating ApoB, whereas ApoB48 is confined to chylomicrons (CM) and only ~0.1% of total ApoB (38, 39).

ApoB100-containing particles (VLDL remnants and LDL) follow traditional LDL-C pathways once deposited in the arterial intima: Binding to proteoglycans via ApoB100’s basic amino acid clusters leads to retention, oxidative modification of ApoB100 lysine residues, and generation of oxidized lipoproteins—mechanistically identical to classic LDL oxidation (40). The resulting ox-LDL-like particles engage scavenger receptors on monocytes and macrophages, triggering foam cell formation and activation of NF-κB–mediated inflammatory signaling (19). This dovetails with the traditional pathway in which elevated LDL-C primes macrophages to produce proinflammatory cytokines (IL-1β, IL-6, TNF-α) and chemokines (MCP-1), thereby accelerating atherogenesis (22).

**Figure 1:**
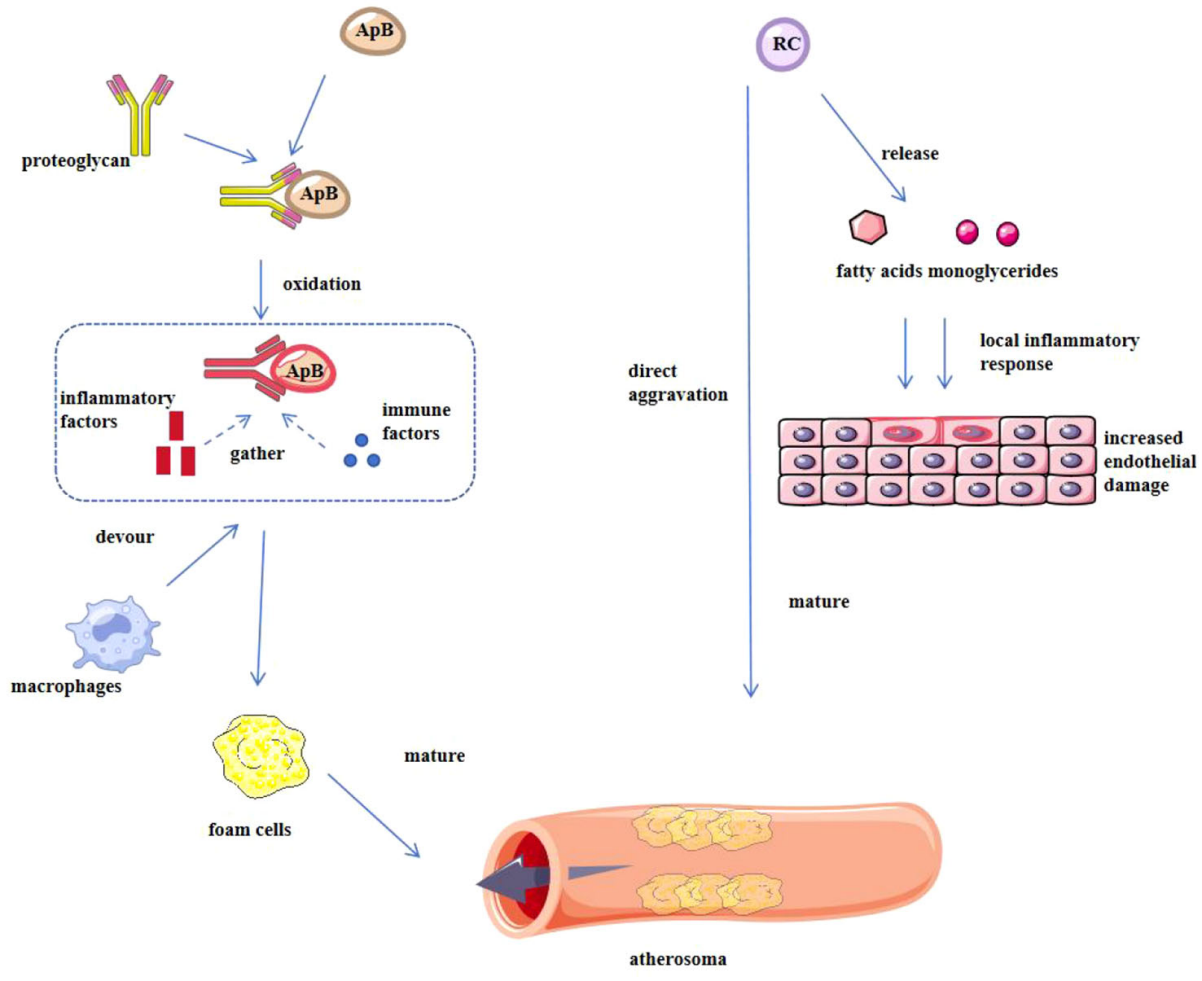
Mechanism of action of ApoB and RC in causing atherosclerosis. ApoB binds to endothelial proteoglycans, leading to lipoprotein retention and oxidation, inducing an inflammatory response, and attracting monocytes to differentiate into macrophages, which in turn transform into foam cells, marking atherosclerosis; RC not only directly causes atherosclerosis, but also releases free fatty acids and monoglycerides through surface lipoprotein lipase, induces endothelial inflammation, aggravates vascular damage, and contributes to the development and progression of atherosclerosis together with other factors.

Moreover, because VLDL remnants serve as precursors to LDL, increased ApoB100 levels reflect augmented hepatic VLDL production—a process regulated by SREBP-2 feedback in the classical cholesterol synthesis pathway (18). Under conditions of high hepatic cholesterol (traditional LDL feedback), LDL receptor (LDLR) activity is downregulated, pushing more cholesterol-rich VLDL into circulation. Thus, ApoB measurement integrates both traditional cholesterol synthesis/clearance feedback and residual VLDL‐derived particle burden. Elevated ApoB amplifies the traditional inflammatory cascade: Oxidized ApoB100 triggers Toll-like receptor 4 (TLR4)–MyD88 signaling in macrophages, synergizing with classical oxidized LDL–induced NLRP3 inflammasome activation (27) **Figure 1**.

As the inflammatory response progresses, smooth muscle cells migrate to the intima under the influence of growth factors (PDGF, TGF-β), synthesize extracellular matrix proteins, and contribute to fibrous cap formation—again mirroring traditional LDL-C–mediated smooth muscle activation (6). In diabetes or obesity, ApoB’s superiority over Non-HDL-C (ESC/EAS 2021) arises from its ability to quantify particle number, which directly correlates with the extent of these shared mechanistic pathways, whereas Non-HDL-C only reflects cholesterol mass and may underestimate risk when cholesterol-enriched particles carry less cholesterol per particle (e.g., small dense LDL) (41).

### 4.3 Lp(a)

Lipoprotein(a) [Lp(a)] is a unique macromolecular complex consisting of a low-density lipoprotein (LDL) particle covalently linked to apolipoprotein(a) [apo(a)] via disulfide bonds (42–44). This structural composition allows Lp(a) to engage both traditional cholesterol-driven atherogenic pathways and prothrombotic cascades, conferring a dual pathophysiological role. The LDL backbone of Lp(a) undergoes oxidation upon arterial deposition, following the classical LOX-1–mediated uptake and foam cell–driven inflammatory cascade observed in LDL-C–induced atherogenesis (19). Simultaneously, the apo(a) component binds with high affinity to plasminogen receptors—structurally homologous to fibrinogen—thereby competitively inhibiting fibrinolysis and obstructing plasmin generation. This mirrors oxidized LDL’s upregulation of PAI-1 in endothelial cells, reinforcing a prothrombotic phenotype common to advanced atherosclerotic plaques (36, 45).

Genetically elevated Lp(a) thus mediates a two-pronged mechanism: First, oxidation of its ApoB100 core produces ox-Lp(a), which activates macrophage scavenger receptors (SR-A, CD36), initiating foam cell formation and secretion of pro-inflammatory cytokines (IL-1β, IL-6, TNF-α)—a pathway converging with oxidized LDL–induced NLRP3 inflammasome activation (19, 27). Second, apo(a) competitively binds fibrinogen and plasminogen, attenuating fibrinolytic cleavage and amplifying thrombotic risk (45). Furthermore, oxidized Lp(a) generates oxidation-specific epitopes (OSEs) that engage Toll-like receptor 2/4 (TLR2/4), enhancing inflammatory signaling and endothelial adhesion molecule expression (VCAM-1, ICAM-1) (46).

Beyond promoting early lesion formation via LDL-like oxidation and inflammation, Lp(a) also disrupts vascular remodeling. Apo(a) displaces plasminogen from endothelial surfaces, akin to LDL-C–induced endothelial dysfunction through reduced eNOS phosphorylation (26). By inhibiting plasmin, Lp(a) prolongs extracellular matrix degradation and delays fibrous cap stabilization, a process analogous to matrix metalloproteinase (MMP) activation in oxidized LDL–exposed smooth muscle cells (6). This combination of early atherogenesis and late-stage plaque destabilization underscores Lp(a)’s multifaceted contribution to cardiovascular disease.

**Figure 2:**
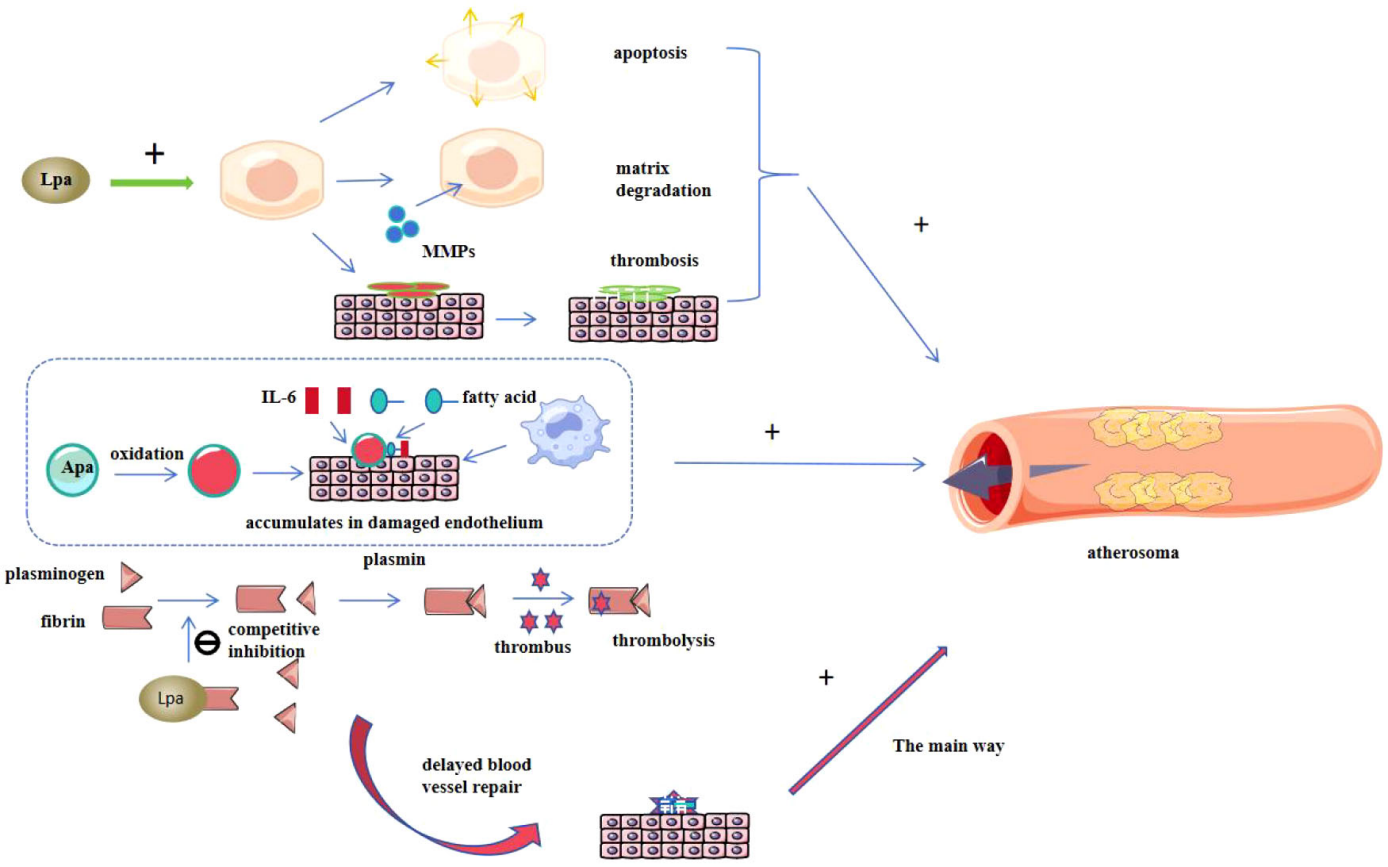
Proposed mechanism by which Lp(a) promotes atherogenesis. Lipoprotein (a) accelerates apoptosis, matrix degradation, and thrombosis, thereby promoting lesion development; apolipoprotein (a) (a component of lipoprotein a) binds to smooth muscle cells and vascular endothelial cells, resides in damaged endothelium, attracts macrophages and monocytes, and participates in atherosclerosis; Lipoprotein (a) competitively inhibits fibrinogen activity, interfering with fibrinogen, fibrin, binding of endothelial cells and extracellular matrix, reducing thrombolysis and delaying vessel wall repair, thus accelerating the progression of atherosclerosis.

Taken together, these overlapping mechanisms position Lp(a) as both an independent risk factor and a pathophysiological amplifier of LDL-C–driven atherosclerosis and thrombosis, highlighting its therapeutic potential as a dual-target for anti-inflammatory and antithrombotic intervention (47–50) **Figure 2**.

### 4.4 Residual cholesterol

Triglyceride-rich lipoproteins (TRLs)—including chylomicron remnants and VLDL remnants—yield residual cholesterol (RC) after partial metabolic clearance (51, 52). In contrast to classical LDL-C which must undergo oxidative modifications (via LOX-1 or scavenger receptors) to become foam-cell inducers, RC can exert direct atherogenic effects without first converting into ox-LDL. Mechanistically, RC particles penetrate the arterial intima similarly to LDL-C, but once retained, their phospholipid core and ApoB residues are directly recognized by macrophage scavenger receptors, accelerating foam cell formation via a pathway that parallels but is independent of traditional LDL-C oxidation (19, 53).

Furthermore, RC-derived free fatty acids (FFA) and monoacylglycerols—by-products of lipoprotein lipase (LPL) hydrolysis—activate Toll-like receptor 4 (TLR4) on endothelial cells, inducing NF-κB–mediated transcription of adhesion molecules (VCAM-1, ICAM-1) and proinflammatory cytokines (IL-1β, IL-6, TNF-α), thereby mirroring the endothelial dysfunction seen in oxidized LDL-C–mediated eNOS uncoupling (53–55). In this way, RC directly amplifies the same proinflammatory circuits central to traditional LDL-driven atherogenesis.

In hypertriglyceridemic states, elevated RC levels lead to downregulation of LDL receptor (LDLR) activity via hepatic feedback (SREBP-2), echoing the classical mechanism by which high intracellular cholesterol suppresses LDL-C clearance (41, 54). As a result, more VLDL (and thus more RC) enters circulation, compounding both LDL-C and RC loads in the arterial wall.

RC also exerts prothrombotic effects: its remnants can bind to fibrinogen and inhibit plasminogen activation—similar to Lp(a) and ox-LDL—thereby reinforcing the traditional thrombotic cascade observed in advanced atherosclerotic plaques (36, 45). RC’s synergy with oxidized LDL-C and small dense LDL amplifies reactive oxygen species (ROS) generation and NLRP3 inflammasome activation, leading to more rapid plaque progression and increased risk of rupture—mechanisms that overlap with the classical LDL-C–induced inflammatory-thrombotic axis (26, 27, 56).

Under certain pathophysiological conditions (e.g., obesity, insulin resistance), RC’s atherogenic potency may exceed that of LDL-C due to its dual capacity to provoke both inflammation and thrombosis without requiring preliminary oxidation (53) **Figure 1**.

## 5 Epidemiologic studies of the association between nontraditional lipids and ASCVD risk

### 5.1 Non-HDL-C

Non-HDL-C levels are significantly associated with the risk of cardiovascular disease (CVD), as demonstrated by multiple studies. Brunner et al. reported a positive correlation between elevated Non-HDL-C concentrations and an increased incidence of CVD in both males and females (57). Individuals with Non-HDL-C levels exceeding 2.6 mmol/L exhibited a substantially higher risk of CVD compared to those with lower concentrations. Supporting this, Wu et al. found that persistently elevated Non-HDL-C levels further increased the risk of CVD in hypertensive patients (58). Moreover, Hansen et al. demonstrated a strong positive association between Non-HDL-C concentrations and all-cause mortality, highlighting that elevated Non-HDL-C levels were linked to increased mortality, even among individuals with well-controlled LDL-C (59). Notably, Wu et al. identified a correlation between elevated childhood levels of LDL-C and Non-HDL-C and the risk of both fatal and non-fatal atherosclerotic cardiovascular disease (ASCVD) events in adulthood (60). Importantly, Non-HDL-C exhibited superior predictive performance compared to LDL-C in assessing ASCVD risk, underscoring its enhanced accuracy and clinical utility.

Given these findings, Non-HDL-C is not only a robust marker of CVD risk but also surpasses LDL-C in predicting long-term cardiovascular events and all-cause mortality. Consequently, Non-HDL-C should be considered a key parameter in cardiovascular risk assessment and cholesterol management in clinical practice.

### 5.2 ApoB

Apolipoprotein B (ApoB) plays a crucial role in assessing cardiovascular disease (CVD) risk. Johannesen et al. demonstrated in the Copenhagen General Population Study that elevated ApoB levels were superior to low-density lipoprotein cholesterol (LDL-C) in predicting the risk of atherosclerotic cardiovascular disease (ASCVD) (61). ApoB levels were found to be dose-dependently associated with an increased risk of myocardial infarction and ASCVD. Similarly, Yun et al. showed that while high LDL-C and low ApoB concentrations did not significantly elevate risk, individuals with low LDL-C but high ApoB had a higher risk of ASCVD (62). This suggests that measuring ApoB levels may provide additional critical information for ASCVD risk assessment, even in individuals with normal LDL-C levels.

However, a prospective study by Su et al. found that although high ApoB levels (without a corresponding significant increase in LDL-C or Non-HDL-C) were associated with a higher risk of CVD, the inclusion of ApoB in existing atherosclerotic risk scores offered limited improvement in risk prediction, particularly in the Chinese population (63). This highlights that, while ApoB is strongly linked to CVD risk, its incremental predictive value may not significantly enhance current risk assessment models. Furthermore, a cohort study by Dong et al. identified a paradoxical finding: individuals with ApoB levels below 0.7 g/L had a higher risk of coronary heart disease compared to those with ApoB ≥ 0.7 g/L (P < 0.05) (64). This finding contradicts previous research, suggesting that lower ApoB levels may also be associated with an increased risk of cardiovascular disease. The underlying mechanisms warrant further investigation. Plasma ApoB concentrations are generally comparable across racial groups, but African Americans have higher levels of oxidized phospholipids per ApoB particle compared to Whites or Hispanics, potentially indicating a greater burden of atherogenic particles in this population (65).

In conclusion, while ApoB serves as a direct marker of atherogenic lipoprotein particles and holds substantial potential in cardiovascular risk assessment, its clinical application requires further research to establish optimal thresholds and refine its predictive accuracy.

### 5.3 Lp(a)

Elevated levels of lipoprotein(a) [Lp(a)] are strongly associated with an increased risk of atherosclerotic cardiovascular disease (ASCVD), as demonstrated by multiple studies. In the Cardiovascular Risk Study by Raitakari et al. elevated Lp(a) levels in adolescence were identified as an independent risk factor for ASCVD in adulthood, highlighting the predictive value of Lp(a) for early cardiovascular risk (66). Similarly, Patel et al. found a linear relationship between Lp(a) levels and ASCVD risk in a cohort from the UK Biobank, reinforcing the notion that higher Lp(a) concentrations correspond to elevated ASCVD risk (67). This underscores the potential of Lp(a) as a valuable marker for ASCVD risk prediction in both primary and secondary prevention settings.

Furthermore, a retrospective cohort study by Berman et al. confirmed that elevated Lp(a) is independently associated with the long-term risk of major adverse cardiovascular events (MACE) (68). However, the study also indicated that the thresholds for risk assessment may vary between primary and secondary prevention populations, suggesting that Lp(a)’s clinical utility should be further evaluated within different prevention contexts. Kaiser et al. found no significant difference in coronary artery disease severity or plaque load between high and low Lp(a) groups (69). However, patients with elevated Lp(a) exhibited faster progression of low-attenuation, vulnerable plaques, which could contribute to an increased ASCVD risk. This suggests that elevated Lp(a) may exacerbate ASCVD risk by promoting the development and progression of vulnerable plaques. The commonly used threshold for elevated Lp(a) levels is ≥50 mg/dL or ≥125 nmol/L. The U.S. National Lipid Association defines Lp(a)-related risk levels as low (<30 mg/dL or <75 nmol/L), moderate (30–49 mg/dL or 75–124 nmol/L), and high (≥50 mg/dL or ≥125 nmol/L) (70).The European Society of Cardiology and European Atherosclerosis Society define extremely high Lp(a) levels as ≥180 mg/dL or 430 nmol/L, which corresponds to the 99th percentile, and is associated with a cardiovascular risk comparable to familial hypercholesterolemia (41).Moreover, multiple large-scale independent studies have identified significant racial and ethnic differences in plasma Lp(a) concentrations and population distribution. Among all racial and ethnic groups studied, Lp(a) levels are highest in Black individuals, followed by South Asians, Whites, Hispanics, and East Asians. Overall, elevated Lp(a) levels are associated with increased cardiovascular risk across all populations (71).

In conclusion, Lp(a) likely contributes to ASCVD risk through multiple mechanisms, including the accelerated progression of susceptible plaques. As such, Lp(a) should be considered an essential adjunct in cardiovascular risk assessment, particularly for identifying high-risk individuals in primary and secondary prevention.

### 5.4 Residual cholesterol

A significant association between remnant cholesterol (RC) levels and atherosclerotic cardiovascular disease (ASCVD) risk has been established. Yang et al. demonstrated that distinct RC trajectories are significantly correlated with vascular endothelial dysfunction and the progression of atherosclerosis (72). Notably, a sustained increase in RC levels may enhance early risk identification for cardiovascular disease (CVD), highlighting its potential role in early risk stratification. Quispe et al. further reported that elevated RC levels are independently associated with ASCVD risk, irrespective of LDL-C, ApoB, and traditional risk factors, underscoring RC as an independent risk determinant (73).

Moreover, Delialis et al. identified both linear and non-linear associations between RC levels and the risk of major adverse cardiovascular events (MACE), a relationship validated in both diagnosed and undiagnosed ASCVD patients (74). This finding underscores the predictive utility of RC in both primary and secondary prevention. Additionally, Chen et al. observed a linear association between RC and CVD risk, alongside a non-linear relationship with coronary heart disease (CHD) risk (75). Specifically, RC levels below 0.84 mmol/L were associated with stable risk, whereas levels exceeding this threshold correlated with a marked risk escalation, particularly among overweight or diabetic individuals. Moreover, the strong association between RC and cardiovascular disease has been confirmed in multi-ethnic studies (76).

RC has been recognized as a robust predictor of MACE and an independent risk factor for ASCVD (77). However, despite its established significance in risk assessment, optimal treatment thresholds and effective strategies for RC reduction remain unclear. Further research is warranted to determine the ideal RC target levels and elucidate the underlying mechanisms mediating its role in CVD prevention.

## 6 Therapeutic measures for non-traditional lipoproteins

The primary target for managing dyslipidemia remains LDL-C, with statins being the first-line therapy for LDL-C reduction (78). Statins exert their effect by inhibiting 3-hydroxy-3-methylglutaryl coenzyme A (HMG-CoA) reductase in the liver, thereby reducing cholesterol synthesis. This leads to a decrease in serum levels of ApoB, LDL-C, and triglycerides (TG), as well as an upregulation of the low-density lipoprotein receptor (LDLR) on hepatocyte surfaces, enhancing the uptake and clearance of circulating LDL-C. However, residual cardiovascular risk persists in some individuals, even after statin therapy has successfully reduced LDL-C. Recent advances in lipid metabolism research have led to the development of new therapeutic agents that target non-traditional lipid parameters. For example, drugs such as ezetimibe, angiopoietin-like protein 3 (ANGPTL3) inhibitors, and Protein Convertase Subtilisin/Kexin Type 9(PCSK9)inhibitors have emerged as promising options for addressing these residual lipid-related risks (79).

### 6.1 PCSK9 inhibitors

**Figure 3:**
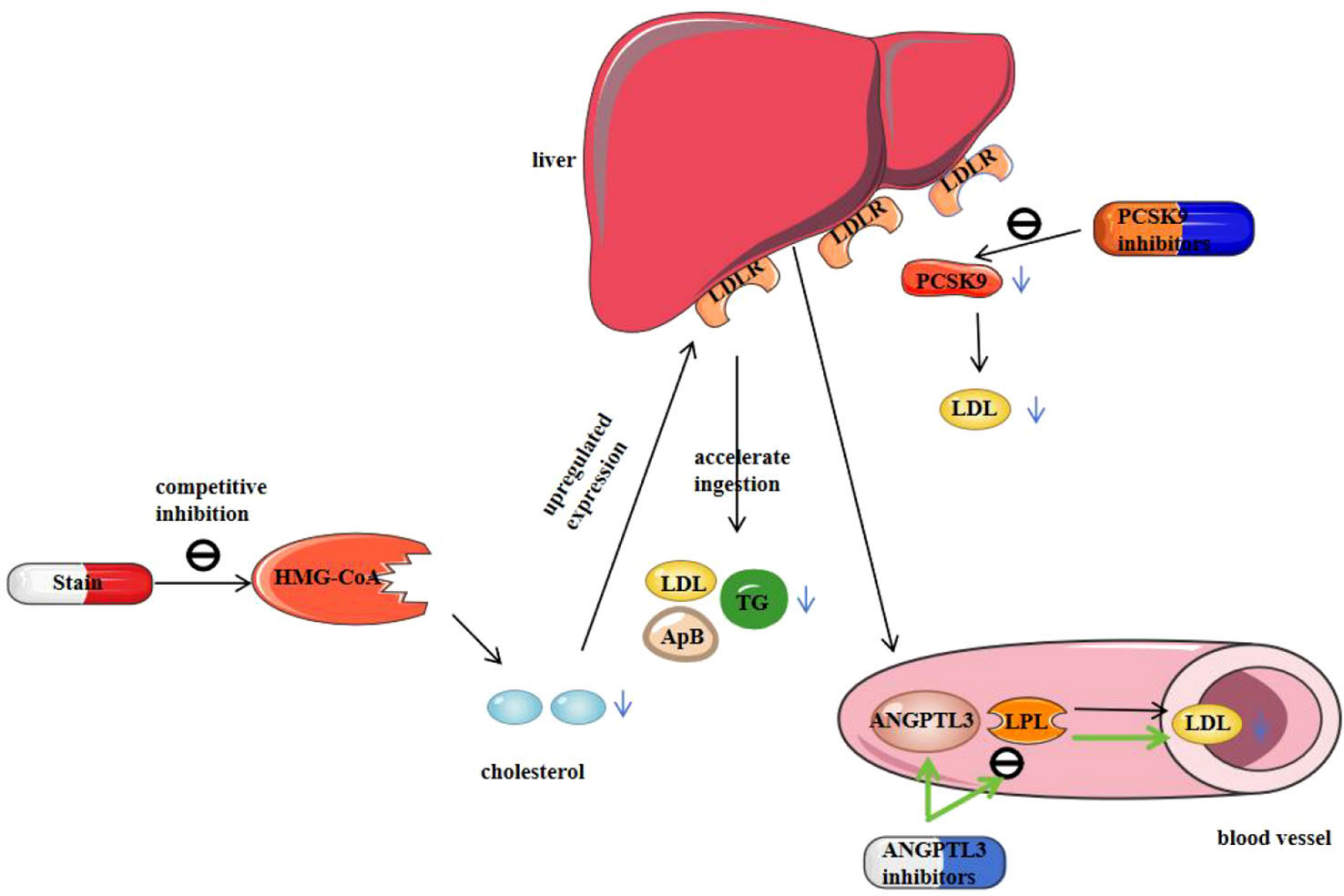
Mechanism of action of statins, PCSK9 inhibitors, and ANGPTL3 inhibitors. Statins competitively inhibit HMG-CoA reductase and reduce cholesterol synthesis, thereby increasing LDL-cholesterol receptor expression, improving LDL-cholesterol uptake, and lowering serum Apo B, LDL-cholesterol, and triglyceride levels. PCSK9 inhibitors effectively reduce LDL-C levels by lowering the plasma concentration of PCSK9 and upregulating LDL-cholesterol receptor expression. ANGPTL3 inhibitors can alleviate the inhibitory effect of ANGPTL3 on LPL, promote fatty acid metabolism, and reduce blood lipid levels.

PCSK9 is a protein produced by the liver, which can bind to low-density lipoprotein receptors (LDLR) on the surface of hepatocytes, promoting the degradation of LDLR, thereby reducing the clearance of LDL-C. In addition, PCSK9 can also promote the synthesis and secretion of VLDL–ApoB through multiple mechanisms, including stabilizing ApoB100, enhancing microsomal triglyceride transfer protein (MTP) activity, and activating the SREBP2 pathway, facilitating the production and release of VLDL–ApoB, leading to elevated levels of atherogenic lipoproteins in plasma (80). PCSK9 inhibitors, as novel lipid-lowering agents, primarily include two types: monoclonal antibodies and small interfering RNA (siRNA). Studies have shown that PCSK9 inhibitors can significantly reduce low-density lipoprotein cholesterol (LDL-C) levels by 50%-60%, as well as reduce lipoprotein(a) [Lp(a)] levels by 25%-30% and triglyceride (TG) levels by 10%-20% (81). Among them, monoclonal antibody-based PCSK9 inhibitors, such as Alirocumab, Evolocumab and Tafolecimab., work by binding to PCSK9, thereby lowering the concentration of PCSK9 in plasma. This reduces PCSK9-mediated degradation of low-density lipoprotein receptors (LDLR), upregulates LDLR expression on hepatocyte surfaces, and ultimately leads to a significant reduction in LDL-C levels (82–84). On the other hand, a representative siRNA-based PCSK9 inhibitor is Inclisiran. Its mechanism of action involves binding to complementary sequences of target messenger RNAs (mRNAs), thereby interfering with the expression of specific genes and inhibiting their translation, which reduces the synthesis of PCSK9 (85, 86) **Figure 3**. In the ORION clinical trials, both single- and double-dose groups of Inclisiran showed a decrease in Lp(a) levels, although the reduction did not reach statistical significance, likely due to individual variability. However, Inclisiran demonstrated statistically significant reductions in non-high-density lipoprotein cholesterol (Non-HDL-C), total cholesterol (TC), and apolipoprotein B (ApoB) levels (87–89). Inclisiran’s unique advantage lies in its long duration of action, requiring only two doses per year to achieve sustained and significant reductions in LDL-C levels. Additionally, it has a favorable safety profile, with no serious adverse events reported, highlighting its substantial potential for clinical use. The ORION-4 trial is expected to enroll 15,000 ASCVD patients with a 5-year follow-up to evaluate the impact of Inclisiran on major adverse cardiovascular events; however, results are not anticipated until around 2026.

### 6.2 Ezetimibe

**Figure 4:**
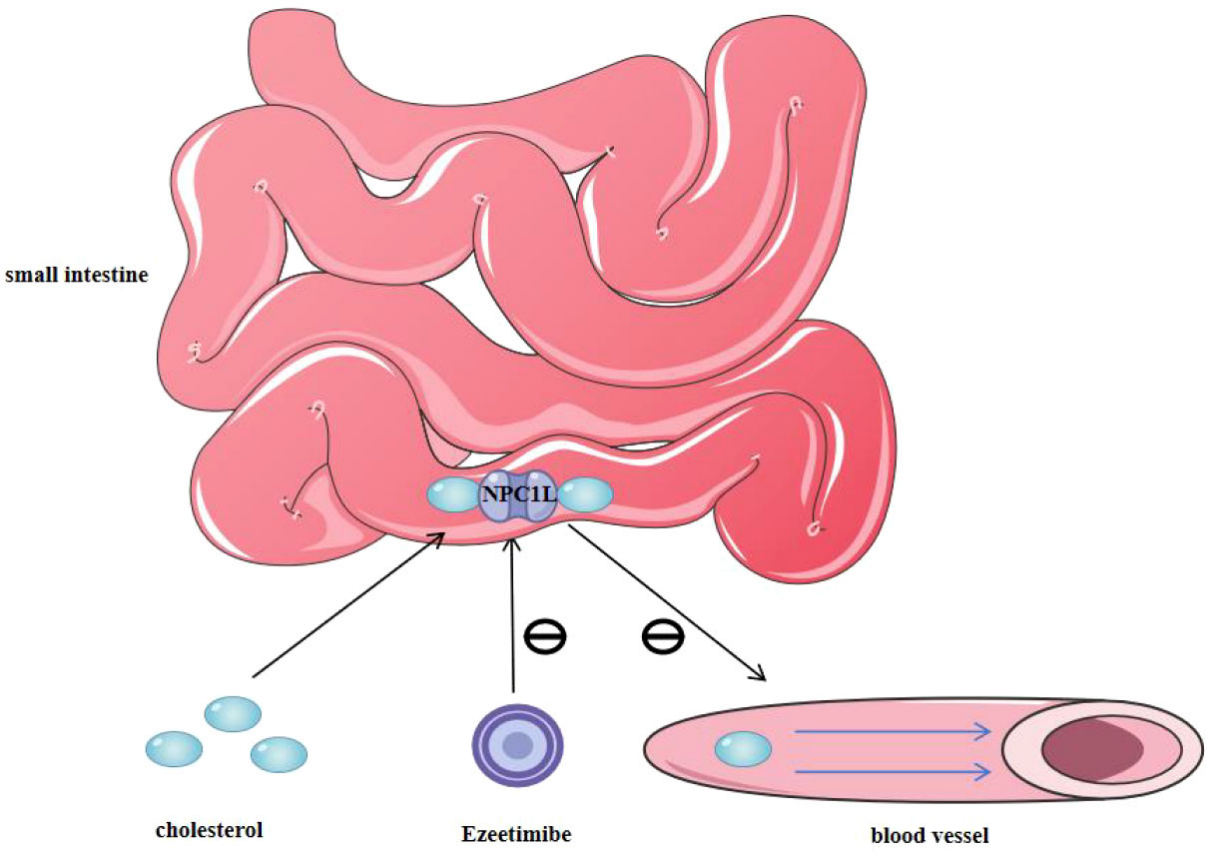
The mechanism of action of ezetimibe. Ezetimibe selectively reduces small intestinal cholesterol absorption by inhibiting NPC1L1 protein.

Ezetimibe, a selective inhibitor of cholesterol uptake, disrupts the function of the Niemann-Pick C1-like 1 (NPC1L1) protein, specifically preventing the absorption of cholesterol in the small intestine without affecting the absorption of other fat-soluble vitamins (90–92). NPC1L1, located on the brush border of the small intestine, plays a crucial role in the uptake of cholesterol. Ezetimibe binds to this protein, inhibiting the transport of cholesterol from the lumen into the intestinal epithelial cells, thereby reducing cholesterol absorption and enhancing its excretion **Figure 4**. Studies have demonstrated that combining ezetimibe with statins significantly improves lipid profiles compared to statin monotherapy. This combination therapy reduced triglycerides (TG) by 12%, apolipoprotein B (ApoB) by 14%, and low-density lipoprotein cholesterol (LDL-C) by an additional 24%, beyond the effects of statins alone (93). This synergistic lipid-lowering effect not only enhances the efficacy of lipid management but also offers a valuable therapeutic option for patients who are intolerant to high-dose statins or have contraindications to statin therapy.

### 6.3 ANGPTL3 inhibitors

In the regulation of lipid metabolism, the liver-specific secreted protein ANGPTL3 inhibits triglyceride (TG) hydrolysis in peripheral capillaries by reducing lipoprotein lipase (LPL) activity. Furthermore, ANGPTL3 inhibition lowers the levels of apolipoprotein B (ApoB), low-density lipoprotein cholesterol (LDL-C), and high-density lipoprotein cholesterol (HDL-C), highlighting its critical role in lipid metabolism (94–96). Inhibiting ANGPTL3 significantly reduces plasma triglyceride levels and lowers Lp(a) levels, a process mediated by pelacarsen, a second-generation hepatocyte-targeted antisense oligonucleotide (97–99). Currently, pelacarsen is undergoing a phase 3 clinical trial, Lp(a) HORIZON (NCT04023552). Preliminary data indicate a significant reduction in Lp(a) concentrations in both single-dose and multiple-dose groups, suggesting that pelacarsen holds considerable potential for lowering Lp(a) levels (100).

Collectively, these emerging therapies—ranging from PCSK9 inhibitors to ANGPTL3 antagonists—represent a significant advance in lipid-lowering strategies beyond LDL-C reduction. They demonstrate distinct mechanisms of action that target residual lipid abnormalities and modulate inflammation and atherogenesis more comprehensively. Such therapeutic expansion offers renewed promise for high-risk patients who fail to reach optimal cardiovascular outcomes with statin monotherapy alone.

However, these novel agents are not without limitations. First, cost and accessibility remain major barriers to widespread clinical implementation, particularly in low- and middle-income populations. PCSK9 monoclonal antibodies and inclisiran, for instance, require long-term administration and are currently unaffordable in many healthcare settings. Second, long-term safety data, especially concerning ANGPTL3 inhibitors and Lp(a)-targeting therapies like pelacarsen, are still under investigation and lack definitive evidence from cardiovascular outcome trials. Third, inter-individual variability in treatment response—possibly due to genetic, metabolic, or inflammatory backgrounds—may limit universal efficacy. Lastly, the integration of these agents into existing treatment algorithms remains uncertain, as optimal sequencing, combination, or substitution strategies have yet to be clearly defined.

Future research should therefore focus on identifying appropriate patient subgroups through biomarker stratification, conducting long-term outcome trials, and evaluating cost-effectiveness across diverse healthcare systems.

## 7 Discussion

As secondary targets in lipid-lowering therapy, the precise mechanisms by which non-traditional lipoproteins contribute to atherogenic plaque formation—particularly in the context of inflammation, thrombosis, and atherosclerosis—require further elucidation. Advancements in detection methodologies are also critical; the development of more cost-effective and clinically applicable assays for non-traditional lipoproteins is imperative. Discrepancies between direct and indirect measurement approaches currently limit the accuracy of risk assessment, underscoring the need for technological optimization or novel assay platforms to enhance reliability and reproducibility. On the therapeutic front, the identification of novel drug targets for non-traditional lipoproteins represents a key research priority. For instance, ANGPTL3 inhibitors have demonstrated promising lipid-lowering potential; however, their long-term safety and clinical efficacy warrant validation through large-scale clinical trials. Nevertheless, significant challenges remain. First, further evidence is required to determine whether non-traditional lipoproteins can independently predict ASCVD risk or complement traditional lipoprotein-based assessments. Second, the protracted timelines and high costs of epidemiological studies hinder data acquisition and analysis. Additionally, the expense and potential adverse effects of emerging therapies may limit their widespread adoption, while the scarcity of clinical data constrains the advancement of precision medicine in this domain.

LDL-C remains the primary therapeutic target in dyslipidemia management and ASCVD prevention, given its well-established role as an independent risk factor. Consequently, most lipid-lowering therapies have been designed to reduce LDL-C levels. However, despite achieving optimal LDL-C control, some individuals continue to experience ASCVD events, highlighting the existence of residual cardiovascular risk. Non-traditional lipid markers—including Lp(a), Non-HDL-C, ApoB, ApoC-III, remnant cholesterol (RC), small dense LDL (sdLDL), and oxidized LDL (oxLDL)—have been strongly associated with this residual risk. Several dyslipidemia management guidelines have already recognized markers such as Non-HDL-C and RC as secondary therapeutic targets to refine treatment strategies. Studies have shown that the apo(a) domain of Lp(a) can bind covalently to LDL particles, leading to deposition and oxidation within the vascular intima, thereby inducing an inflammatory cascade and promoting foam cell formation; In addition, apo(a) competitively inhibits plasminogen activation, enhancing thrombogenic potential. Genetic studies also support its pathogenic potential—multiple Mendelian randomization studies suggest that, high Lp(a) levels are causally associated with increased risk of ASCVD. Therefore, Lp(a) serves not only as a biomarker for predicting cardiovascular risk, but also plays a direct pathogenic role in the development of cardiovascular disease. Overall, non-traditional lipoprotein biomarkers such as Lp(a), ApoB, and ApoC-III, serve as important complements in evaluating residual cardiovascular risk, and their value in clinical risk stratification and personalized intervention is increasingly recognized.

In summary, while LDL-C remains central to lipid management, the assessment of non-traditional lipoproteins offers valuable insights into residual ASCVD risk. For example, given the potential of Lp(a) to enhance early detection and preventive strategies across diverse patient populations, current evidence supports the inclusion of lipoprotein(a) screening in routine cardiovascular risk assessment (101). However, it is important to note that reference ranges and threshold values vary across different racial and ethnic groups, and further studies are needed to validate its generalizability and ethnic-specific relevance, with a greater focus on elucidating the pathogenic mechanisms and therapeutic potential of these biomarkers, thereby advancing comprehensive strategies for cardiovascular disease prevention and treatment.
